# The Optimal Sizing of Substation Capacity in a Distribution Network, Considering a Dynamic Time-of-Use Pricing Mechanism

**DOI:** 10.3390/s22197173

**Published:** 2022-09-21

**Authors:** Dajun Xu, Qiang Fu, Lun Ye, Wenyu Lin, Yuzhou Qian, Kebo Zhang, Jiangang Yao

**Affiliations:** 1 State Grid Ningbo Yinzhou Power Supply Company, Ningbo 315000, China; 2 College of Electrical and Information Engineering, Hunan University, Changsha 410082, China

**Keywords:** time-of-use price, distribution network planning, substation capacity sizing, demand side management, cost-benefit analysis, peak load shifting

## Abstract

Application of sensors in the smart grid has promoted the development of demand side management (DSM). However, the incentives of DSM such as peak–valley time-of-use (TOU) price will change the load pattern in the future; the substation capacity sizing will be further influenced accordingly. This paper proposes a substation capacity sizing method in distribution network considering DSM and establishes a peak-valley TOU pricing method based on the cost–benefit analysis of each participant in the TOU price. Compared with the conventional fixed peak–valley ratio, a dynamic division method is proposed to calculate the optimal pull-off ratio for the TOU pricing. By considering the proposed TOU pricing method, a substation capacity sizing model for the distribution network is further proposed. Finally, the economic benefits of the two substation capacity sizing schemes are compared and evaluated according to the selected economic indicators. The results of the case study demonstrate that under the premise of reasonable pricing, considering the impact of TOU on substation capacity sizing, the construction investment and the user cost of power supply companies can be saved while meeting the power demand. The economy and rationality of the planning scheme have been significantly improved.

## 1. Introduction

With the development of the internet of things and sensors, demand side management (DSM) has been widely implemented in the smart grid. The elasticity of market demand has improved. Power supply companies have more choices in power supply and electricity price strategies for the future, which makes the operation mode of the distribution network more diversified [1,2,3]. A traditional distribution network planning scheme is formulated according to the rigid demand of user load and socioeconomic development [4], in which it is difficult to consider the influence of DSM implementation in the power system and formulate reasonable planning schemes. Therefore, it is necessary to take into account the effect of DSM in distribution network planning to fully improve its feasibility, rationality, and economy [5].

As the most commonly used economic measure of DSM, the peak–valley time-of-use (TOU) price is the main way to reduce the maximum load [6]. The peak–valley TOU price is an important stimulus provided by power supply companies to demand side users through price leverage to implement integrated resource management in the smart grid [7,8]. It divides an operation cycle (day, month, quarter, year, etc.) into three periods: peak, normal, and valley. Based on the price at normal period, the peak–valley TOU price raises the price at peak period and reduces the price at valley period. It can cut the peak and fill the valley, alleviate the peak period of the power supply pressure, and improve the system load rate; the electricity cost of users will also be reduced. Moreover, research in [9,10] shows that the implementation of the peak–valley TOU price can also effectively reduce the transmission loss of electric energy in the distribution network. In this way, the peak–valley TOU price can change the spatial and temporal distribution of the overall regional load by adjusting the load characteristics of users, which can further reduce the electricity quantity that the power supply company needs to supply due to the load growth and, ultimately, affect the overall distribution network planning scheme.

Research and practical experience have demonstrated that the peak–valley TOU price is not only an effective method of DSM but also an important measure for further promoting the sustainable and harmonious development of society, the economy, energy, and the environment [11,12,13]. At present, research on the peak–valley TOU price focuses on three aspects: pricing method, users’ response to the peak–valley TOU price, and the cost-effectiveness of DSM participants. Reference [14] studied the division of peak, normal, and valley periods and their pricing methods and established the pricing model of the electricity price in corresponding periods. References [15,16] optimized the pricing method of the peak–valley TOU price by combining the price of the demand side and supply side. References [17,18,19] obtained the demand response model and its corresponding algorithm by studying the response behavior of the demand side after implementation of the TOU price. In order to make the three DSM participants—i.e., power supply companies, users, and the whole society—benefit from the implementation of the TOU price, reference [20] systematically analyzed the cost–benefit of the three participants and established a practical analysis model.

On the basis of the above, the load demand will change with the implementation of the peak–valley TOU price, which will affect the distribution network planning. Substation capacity sizing is one of the important parts of distribution network planning, which can affect the construction investment, the grid structure, the physical-information coordination based on situation awareness, and the power supply reliability. However, numerous studies of distribution network planning considering the TOU are based on the fixed TOU price, regardless of the pricing and the division of the peak and valley. With the gradual promotion of the peak–valley TOU price, it is necessary to study its dynamic impact on future Smart Grid planning to develop a more reasonable and economic planning scheme. The peak–valley TOU price mainly affects substation capacity sizing by adjusting the maximum load, which cannot change the trend of load development. It can only affect the implementation process of power system construction. Therefore, in this paper, a substation capacity sizing method considering the dynamic peak–valley TOU price is proposed and applied to substation capacity sizing to modify and verify its benefit. In the proposed method, the initial substation capacity sizing scheme is formulated without considering the influence of the peak–valley TOU price. On the basis of the initial sizing scheme, the influence of the dynamic peak–valley TOU price is determined on the basis of data perception of the distribution network to modify the scheme, and cost–benefit analysis is conducted to obtain the final sizing scheme. The results of the case study demonstrate that considering the influence of the dynamic peak–valley TOU price in substation capacity sizing under the premise of a reasonable price setting can save the cost for both power supply companies and users.

## 2. Modeling of the Peak–Valley TOU Price

### 2.1. Dynamic Division of the Peak Valley Period

The reasonable peak–valley time division is the premise for obtaining the corresponding reasonable peak–valley TOU price and achieving the good effect of peak load shifting. In this paper, the time division method in [21] is used to determine the possibility of peak and valley periods at each time point on the daily load curve.

Divide the peak and valley periods for 1 day of 24 h; set *h_i_* as the *i*-th division method in total *n* divisions. The division set of the peak and valley periods can be represented as *H* = {*h_i_*, *i* = 1, 2, 3,..., *n*}. In each division method *h_i_*, use the electricity price models of peak, valley, and normal periods to optimize and compare the results of each division in *H* to select the best method for peak load shifting. The optimization models of the electricity price will be discussed in the next section.

### 2.2. Modeling the TOU Price

Suppose $T_{s}$, $T_{l}$, and $T_{v}$ are the peak, level, and valley periods division in a day, respectively, and that this satisfies $T_{s}+T_{l}+T_{v}=24\;h$. $P_{s}$, $P_{l}$, and $P_{v}$ is the corresponding electricity price of the three periods, respectively. $W_{s}$, $W_{l}$, and $W_{v}$ represent the corresponding electricity consumption of the three periods, respectively. $P_{0}$ and $W_{0}$ represent the electricity price and electricity consumption in the whole day before the implementation of the peak–valley TOU price, respectively. According to the overall goal of DSM implementation, the mathematical model of the peak–valley TOU price is established from the following aspects:

1. Profit of power supply company

Before and after the implementation of the peak–valley TOU price, the electricity sales revenue of the power supply company are $G_{0}=P_{0}W_{0}$ and $G_{1}=P_{s}W_{s}+P_{l}W_{l}+P_{v}W_{v}$, respectively. The average daily reduction in the distribution network construction investment due to the reduction in maximum load after the implementation of the peak–valley TOU electricity price is $G_{t}$. To meet the principle of profit for the power supply company, the following relationship should be satisfied:

(1) $$G_{1}+G_{t}\geq G_{0}$$

2. Benefit of the user side

Suppose $P_{1}$ is the average price after the implementation of the peak–valley time-of-use price, according to the principle of user side benefit:

(2) $$P_{1}\leq P_{0}$$

(3) $$P_{1}=\frac{P_{s}W_{s}+P_{l}W_{l}+P_{v}W_{v}}{W_{s}+W_{l}+W_{v}}$$

3. Cost constraint

The electricity price in the valley period should be greater than its marginal cost:

(4) $$C_{e}\leq P_{v}\leq P_{s}$$

4. Prevention of peak–valley inversion

The peak–valley TOU price has limited regulation ability to load and must be regulated within a certain proportion to prevent the phenomenon of peak–valley inversion with excessive regulation, which is not conducive to the safe and stable operation of the power system. Thus, the regulating capability of the peak–valley TOU price should satisfy:

(5) $$-F_{\max}\leq \frac{L_{hi}(P_{s},P_{l},P_{v},t)-L_{0}\left(t\right)}{L_{0}\left(t\right)}\leq F_{\max}$$

where $F_{\max}$ is the maximum adjustable proportion of load, determined by the power supervision department according to the actual situation of the region. $L_{0}\left(t\right)$ and $L_{hi}(P_{s},P_{l},P_{v},t)$ are the load values before and after the implementation of the peak–valley TOU price, respectively.

The main purpose of implementing DSM is to cut the peak and fill the valley, so that the peak–valley difference in the daily load curve is smaller. On the basis of the change and distribution of the electricity price before and after the implementation of the peak–valley TOU price, the optimal peak–valley TOU price model based on the division method *h_i_* is proposed as follows:

$$obj.\;\min\left[\underset{0\leq t\leq 24}{\max}L_{hi}(P_{s},P_{l},P_{v},t)\right]$$

$$\min\left[\underset{0\leq t\leq 24}{\max}L_{hi}(P_{s},P_{l},P_{v},t)-\underset{0\leq t\leq 24}{\min}L_{hi}(P_{s},P_{l},P_{v},t)\right]$$

$$s.t.\;G_{1}+G_{t}\geq G_{0}$$

$$P_{1}\leq P_{0}$$

$$-F_{\max}\leq \frac{L_{hi}(P_{s},P_{l},P_{v},t)-L_{0}\left(t\right)}{L_{0}\left(t\right)}\leq F_{\max}$$

where $\underset{0\leq t\leq 24}{\max}L_{hi}(P_{s},P_{l},P_{v},t)$ and $\underset{0\leq t\leq 24}{\min}L_{hi}(P_{s},P_{l},P_{v},t)$ are the maximum and minimum load of the daily load curve $L_{hi}$ under the peak–valley division method *h_i_*, respectively. $\underset{0\leq t\leq 24}{\max}L_{hi}(P_{s},P_{l},P_{v},t)-\underset{0\leq t\leq 24}{\min}L_{hi}(P_{s},P_{l},P_{v},t)$ is the peak–valley difference in the daily load curve $L_{hi}$.

## 3. Cost–Benefit Analysis and Electricity Pricing

### 3.1. Cost–Benefit Analysis

The power supply company, the user, and government constitute the three main participants in the implementation of the peak–valley TOU price [22]. The power supply company is the main body that implements the electricity price strategy. The user is the implementation object of the electricity price strategy, who gives feedback to the power supply company through DSM. The government is responsible for leading and supervising the implementation process of the electricity pricing strategy, which reflects the benefits of the whole society [23]. Therefore, to analyze the cost–benefit of peak–valley TOU price implementation, it is necessary to take into account its different impacts on the power supply company, on the user, and on government (i.e., the whole society). The interests of the user side need to be secured to facilitate their voluntary participation in DSM [24]. Moreover, in order to obtain government approval, it is also necessary to ensure that the implementation of the electricity price strategy can benefit the whole society.

Combined with the dynamic peak–valley TOU price model established above, this section analyzes the cost, income, and profit variation in the power supply company, the user, and the whole society for a typical load day in distribution network planning. Set $\Delta C_{p}$, $\Delta B_{p}$, and $\Delta E_{p}$ as the changes of cost, profit, and the profit of the power supply company before and after the implementation of the peak–valley TOU price, respectively. $\Delta C_{u}$, $\Delta B_{u}$, and $\Delta E_{u}$ are the changes of cost, profit, and profit of user before and after the implementation of the peak–valley TOU price, respectively. $\Delta C_{s}$, $\Delta B_{s}$, and $\Delta E_{s}$ are the changes of cost, profit, and profit of user before and after the implementation of the peak–valley TOU price, respectively. The cost–benefit analysis is as follows.

#### 3.1.1. Cost–Benefit Changes in the Power Supply Company

Considering the influence of the peak–valley time-of-use price in distribution network planning, the cost change in the power supply company consists of reduced construction investment. Its value can be determined according to the average cost of reduced or postponed construction substation equipment, transmission, and distribution network frame and its supporting facilities. The loss of electricity sales profit caused by the implementation of the peak–valley TOU electricity price constitutes the change in revenue in the power supply company. Therefore, the cost–benefit changes model is:

(6) $$\Delta C_{p}=-G_{t}$$

(7) $$\Delta B_{p}=\left(P_{1}-P_{0}\right)W_{0}$$

where $G_{t}=c_{t}\Delta S_{m}/365ny$, $c_{t}$ is the substation capacity cost (10^6^ RMB/MW). $\Delta S_{m}$ is the difference in new substation capacity between the traditional distribution network planning and the planning considering DSM measures. ny is the number of years in the planning cycle. $W_{0}$ represents the electricity consumption of the whole day before the peak–valley TOU price is implemented. The user adjusts the electricity consumption period after the peak–valley TOU price is implemented, but the normal production activities demand of the user should still be guaranteed, so that the user’s electricity consumption can be assumed to be unchanged.

#### 3.1.2. Cost–Benefit Changes in the User

The change in the user’s electricity cost is the change in electricity payment caused by the change in the average electricity price after the implementation of the peak–valley TOU price (ignoring the change in cost caused by the adjustment of the production process) [25]. The implementation of the peak–valley TOU price in distribution network planning has little effect on user income, so the change in user income is ignored. The cost–benefit change model is:

(8) $$\Delta C_{u}=\left(P_{1}-P_{0}\right)W_{0}$$

(9) $$\Delta B_{u}=0$$

#### 3.1.3. Cost–Benefit Changes in the Whole Society

Reduced investment by the power supply company after taking into account the impact of the peak–valley TOU price in distribution network planning constitutes the cost change in the whole society. The implementation of the peak–valley TOU price in distribution network planning has little effect on the benefits of the whole society, so the change associated with this is ignored. The corresponding cost–benefit changes model is:

(10) $$\Delta C_{s}=-G_{t}$$

(11) $$\Delta B_{s}=0$$

#### 3.1.4. Benefit Analysis of the Three Participants

From Equations (6) to (11), it can be obtained that the profit changes in the three participants after considering the implementation of the peak–valley TOU price in distribution network planning is:

(12) $$\left\{ \begin{array}{l} \Delta E_{p}=\Delta B_{p}-\Delta C_{p}\\ \Delta E_{u}=\Delta B_{u}-\Delta C_{u}\\ \Delta E_{s}=\Delta B_{s}-\Delta C_{s} \end{array}\right.$$

### 3.2. Peak–Valley TOU Pricing

**Figure 1:**
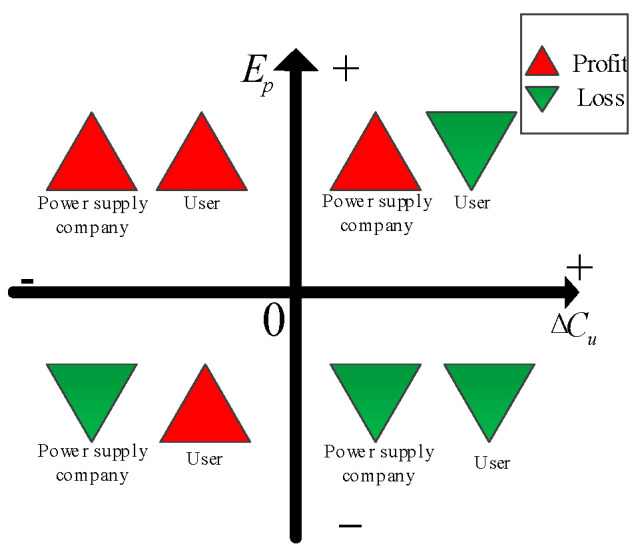
Changes in user electricity cost and power supply company electricity sales profit after DSM implementation.

Figure 1 shows the calculation results of changes in user electricity cost and power supply company electricity sales profit after DSM implementation in the form of a rectangular coordinate diagram. It can be seen from the figure that when the calculation results are in quadrant II, the corresponding DSM measures are beneficial to both the user and the power supply company. When the calculation results are in quadrant IV, it is unfavorable to both the user and the power supply company. When the calculation results are in quadrants I or III, the corresponding DSM measures are only beneficial to either the power supply company or the user.

The implementation of the peak–valley TOU price based on DSM should not only ensure that the power supply company obtains more profits, but also ensure that the user’s electricity expenditure is reduced. In other words, to improve the enthusiasm of users to participate in DSM, the calculation results of changes in user electricity cost and power supply company electricity sales profit after DSM implementation should be in quadrant II of Figure 1. Therefore, it is necessary to set a reasonable peak–valley TOU price to ensure the profit of both the power supply company and the user. The setting of the peak–valley TOU price is analyzed as follows.

#### 3.2.1. Pricing in a Normal Period

Because the price in a normal period represents the overall electricity consumption level in a region, this should not be affected by the implementation of the peak–valley TOU price. Therefore, it can be assumed that the price in a normal period is equal to the average price before the implementation of the peak–valley TOU price.

(13) $$P_{l}=P_{0}$$

#### 3.2.2. Pricing in Peak and Valley Periods

Set the fluctuation range of peak and valley electricity price relative to the normal period as $\delta_{s}$ and $\delta_{v}$, respectively. The corresponding electricity price is

(14) $$P_{s}=P_{0}\left(1+\delta_{s}\right)$$

(15) $$P_{v}=P_{0}\left(1-\delta_{v}\right)$$

Define pull-off ratio β as the ratio of peak and valley electricity price to the fluctuation of price in the normal period; that is,

(16) $$\beta =\frac{\delta_{s}}{\delta_{v}}$$

Then, the following relationship for the electricity price of the user can be depicted:

(17) $$P_{1}=P_{0}\left(1+\frac{\delta_{s}W_{s}-\delta_{v}W_{v}}{W_{s}+W_{l}+W_{v}}\right)$$

(18) $$\frac{P_{1}}{P_{0}}=1+\frac{\delta_{s}W_{s}-\delta_{v}W_{v}}{W_{s}+W_{l}+W_{v}}$$

It can be seen from Equation (18) that to reduce the average price of the user, the value of the pull-off ratio needs to satisfy β<WvWs.

The investment cost of distribution network construction in the power supply company has the following relationship: $G_{t}\propto \Delta S_{\max}$, $\Delta S_{\max}=\Delta L_{\max}/\alpha \cos\theta$, and $G_{t}\propto \Delta L_{\max}$, that is

(19) $$G_{t}=a\Delta L_{\max}$$

where $\Delta S_{\max}$ represents the new substation capacity that can be reduced after the implementation of the peak–valley TOU price. $\Delta L_{\max}$ represents the maximum load change before and after the implementation of the peak–valley TOU price. α is the load rate, cosθ is the power factor.

The research on load transfer rate curve in the user response model established in reference [26] shows that

(20) $$\Delta L_{\max}=\lambda_{sv}L_{\max}+\lambda_{sl}L_{\max}$$

where $\lambda_{sv}$ and $\lambda_{sl}$ is the load transfer rate of the peak–valley period and peak–normal period, respectively. They have the following relationship with peak, normal, and valley electricity prices:

(21) $$\lambda_{sv}=k_{sv}\left(P_{s}-P_{v}\right)+b_{sv}=k_{sv}P_{0}\left(\delta_{s}+\delta_{v}\right)+b_{sv}$$

(22) $$\lambda_{sl}=k_{sl}\left(P_{s}-P_{l}\right)+b_{sl}=k_{sl}P_{0}\delta_{s}+b_{sl}$$

where $k_{sv}$ and $b_{sv}$ are the slope and longitudinal intercept of load transfer rate curve in the peak–valley period, respectively. $k_{sl}$ and $b_{sl}$ are the slope and longitudinal intercept of the load transfer rate curve in the peak–normal period, respectively.

According to Equations (19) to (22), the following relationship can be acquired

(23) $$G_{t}=k_{s}\delta_{s}+k_{v}\delta_{v}+m$$

where $k_{s}=aL_{\max}P_{0}(k_{sv}+k_{sl})$, $k_{v}=aL_{\max}P_{0}k_{sv}$, $m=aL_{\max}(b_{sv}+b_{sl})$.

According to Equations (6) to (8), (12), (17), and (23), it can be ascertained that

(24) $$\Delta B_{p}=\left(P_{1}-P_{0}\right)W_{0}=P_{0}\left(\delta_{s}W_{s}-\delta_{v}W_{v}\right)$$

(25) $$\Delta E_{p}=P_{0}\left(\delta_{s}W_{s}-\delta_{v}W_{v}\right)+k_{s}\delta_{s}+k_{v}\delta_{v}+m$$

where $P_{0}$, $k_{s}$, $k_{v}$, and m are fixed values; and $\Delta E_{p}$ and $\Delta C_{u}$ are binary functions of $\delta_{s}$ and $\delta_{v}$; that is,

(26) $$\left\{ \begin{array}{l} \Delta E_{p}=f_{p}\left(\delta_{s},\delta_{v}\right)\\ \Delta C_{u}=f_{u}\left(\delta_{s},\delta_{v}\right) \end{array}\right.$$

If the peak to valley electricity price ratio of the peak–valley TOU price in a region changes little, it can be considered as a fixed value:

(27) $$K=\frac{P_{s}}{P_{v}}=\frac{1+\delta_{s}}{1-\delta_{v}}$$

According to Equations (16) and (27), it can be conducted that

(28) $$\left\{ \begin{array}{l} \delta_{s}=\frac{\beta \left(K-1\right)}{K+\beta }\\ \delta_{v}=\frac{K-1}{K+\beta } \end{array}\right.$$

According to (28), $\Delta E_{p}$ and $\Delta C_{u}$ can be simplified as univariate functions of β:

(29) $$\left\{ \begin{array}{l} \Delta E_{p}=f_{p}\left(\beta \right)\\ \Delta C_{u}=f_{u}\left(\beta \right) \end{array}\right.$$

Based on the above, through Equation (29), the research on the dynamic peak–valley TOU price can be transformed into the analysis of the impact of the pull-off ratio on the cost–benefit of the power supply company, the user, and the whole society.

## 4. Case Study

In this case study, the annual maximum load day of the 110 kV distribution network in a municipal level area is selected as the typical load day to verify the effectiveness of the proposed method. The relevant data are collected from an actually implemented project. Before the implementation of the peak–valley TOU price, the maximum hourly load of a typical day is 7795 MW and the minimum hourly load is 4915 MW. The electricity consumption for the peak, valley, and normal periods is 44,852 MWh, 50,431 MWh, and 54,956 MWh, respectively. The load data are shown in Table 1.

According to the time division method in Section 2.1, the results of the time period division are as follows: peak period: 9:00–11:00, 17:00–21:00; valley period: 23:00–7:00; normal period: the time period other than the peak and valley periods.

### 4.1. Power Demand Forecasting

According to the regional overall development planning and electricity load reporting in the past three years, the spatial load forecasting method can be used to predict the load in the next three years. The long-term newly increased load is forecasted using the load density forecasting method [27]. The forecasting results in the next three years are shown in Table 2.

### 4.2. Setting of the Peak–Valley TOU Price

In the studied region, the average price is 0.59 RMB/kWh before the implementation of the peak–valley TOU price, and the peak–valley price ratio *K* = 3.8. The pull-off ratio β takes 0 as the starting value and takes 7 typical values with an equal interval of 0.25. According to the method in Section 3, the peak–valley TOU price can be calculated under each pull-off ratio. Based on the dynamic peak–valley TOU price model, the load characteristics after implementing the peak–valley TOU price can be simulated to obtain the daily maximum load, the minimum load, and the maximum load transfer rate under different pull-off ratios (the proportion of the transfer of the user’s maximum load to the normal and valley periods to its original value). The coordinates of changes in the user electricity cost and the power supply company electricity sales profit in Figure 1 can also be calculated, as shown in Table 3.

**Figure 2:**
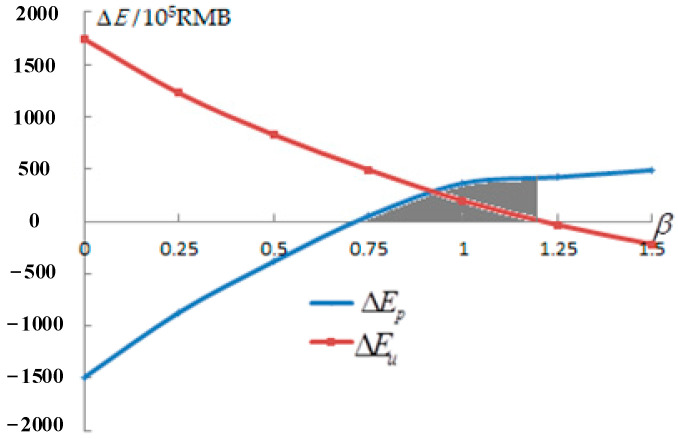
Profit change curve in company and user.

With the change in the pull-off ratio, the profit change curve between the power supply company and the user can be calculated by Equations (6) to (9), and (14), as shown in Figure 2.

According to Table 3 and Figure 2, when the value of β is 0, 0.25, or 0.5, the user gains but the power supply company loses. When the value of β is 1.25 or 1.5, the power supply company gains but the user loses. When the value of β is 0.75 or 1, both the power supply company and the user can benefit. In conclusion, under a constant peak–valley electricity price ratio, if the value of the pull-off ratio is reasonable (The value of β is in the shaded area in Figure 2), the implementation of the peak–valley TOU price will make the profit change curve between the power supply company and the user in quadrant II of Figure 1, so that the power supply company and the user can both profit.

**Figure 3:**
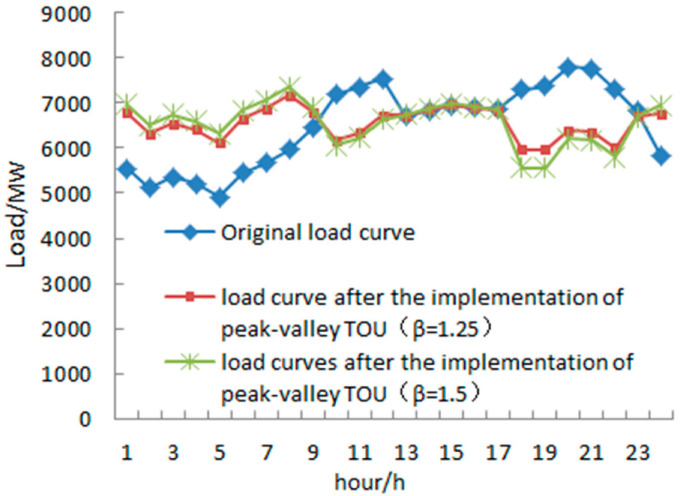
Load curve of a typical load day.

According to the survey of user feedback and relevant historical data after several adjustments in the peak–valley TOU price ratio, combined with the peak–valley TOU price model established in this paper, MatlabR2014a is used as the simulation platform for regression fitting the user feedback after the peak–valley TOU price is adopted. The load curves after the implementation of the peak–valley TOU price are obtained. When the pull-off ratio is 0.75 or 1, the load curves after the implementation of the peak–valley TOU price are shown in Figure 3. According to the figure, after the implementation of the dynamic peak–valley TOU price, the maximum load is significantly reduced, the load rate is increased, and the smoothness of the load curve is improved. When β = 0.75, after implementation of the peak–valley TOU price, the maximum load $L_{\max}$ = 6847 MW, the minimum load $L_{\min}$ = 5631 MW, and the peak valley difference $\Delta L_{sv}$ = 1216 MW. When β = 1, after the implementation of the peak–valley TOU price, the maximum load $L_{\max}$ = 6861 MW, the minimum load $L_{\min}$ = 5851 MW, and the peak valley difference $\Delta L_{sv}$ = 1010 MW. Through comparison, it can be seen that the maximum load difference is small when the pull-off ratio is 0.75 and 1, but the peak valley difference at β = 1 is 206 MW less than that at β = 0.75, and the load curve is smoother. Therefore, the peak–valley TOU price with β = 1 has been selected for this paper to study its impact on substation capacity sizing.

### 4.3. Cost–Benefit Analysis

After the implementation of the dynamic peak–valley TOU price, the peak period electricity consumption of the typical load day is $W_{s}=\int_{Ts}L(T)d_{t}$ = 38,659 MWh. The electricity consumption of the normal period is $W_{l}=\int_{T_{l}}L(T)d_{t}$ = 67,441 MWh. The electricity consumption of the valley period is $W_{v}=\int_{T_{v}}L(T)d_{t}$ = 44,139 MWh. From the Table 3, $P_{s}$ = 0.934 RMB/kWh, $P_{v}$ = 0.246 RMB/kWh, $\Delta C_{u}$ = −153.341 × 10^5^ RMB, $\Delta E_{p}$ = 71.385 × 10^5^ RMB. According to Equations (6) to (12), the cost, income, and profit changes of each participant in the typical load day after considering the implementation of the dynamic peak–valley TOU price in the substation capacity sizing can be calculated. The results are shown in Table 4.

According to Table 4, the reduction in the average electricity price after the implementation of the dynamic TOU results in an income reduction of 1.97567 million yuan, while the temporal shift in the maximum load leads to a reduction in the substation sizing capacity, thus saving 2.69552 million yuan in the investment cost. In the end, the power supply company can still make a profit of 719,850 yuan. Moreover, the reduction in the average electricity price also saves the users 1,533,410 yuan.

### 4.4. Substation Sizing

According to Reference [28], the maximum load transfer rate is only affected by the price difference between the peak and valley periods. Table 3 shows that the maximum load transfer rate in the first year of the past three years is $\lambda_{m}$ = 0.12; the load forecasting results of each year can be modified after considering the effect of the dynamic peak–valley TOU price on the basis of this.

On the basis of the assumption that the same peak–valley TOU price strategy will be implemented in the planning period, the electricity price will remain unchanged. The power demand forecast with β = 1 in the next three years and its correction results considering the impact of DSM implementation are shown in Table 5. Taking into account the current situation of the regional power grid and the annual load growth, the sizing of 110 kV substations in the regional power grid in the next three years is preliminarily determined (assuming that the load rate of the main transformer is 60% and the power factor is 0.9), and the sizing results are modified after considering the implementation of the dynamic peak–valley TOU price, as shown in Table 6.

It can be seen from Table 6 that when taking into account the impact of the dynamic peak–valley TOU price, the new substation capacity required in each year can be effectively reduced. In this way, while meeting the electricity demand, the number of power grid construction projects in the planning cycle is reduced, the planning investment is saved, and the workload of the planners is also reduced. The additional substation capacity in the planning period reduces from 5290 MVA to 4673 MVA.

### 4.5. Investment Cost of Substation

In Table 6, the investment cost of the substation capacity sizing in each year can be estimated according to $G_{p}=c_{t}\Delta S_{m}$ (the unit substation capacity construction cost, take 106 yuan/MVA). The results are shown in Table 7.

As can be seen from Table 7, compared with the traditional planning method, the proposed method has reduced the investment cost in each planning year. The reduction of 100 million yuan to 4.673 billion yuan of the new scheme, saving 11.67% of the investment cost, proves that considering the influence of the dynamic TOU with a reasonable price of electricity in the substation sizing can effectively reduce the investment in power grid expansion and improve the economy of substation capacity sizing.

### 4.6. Verification of the Modified Planning Scheme

Check the power balance of the two substation capacity sizing schemes, and the results are shown in Table 8. According to Table 8, compared with the traditional sizing scheme, the main transformer capacity in each year will be reduced after considering the impact of the dynamic peak–valley TOU price on substation capacity sizing, but its corresponding network supply load will also be reduced due to load transfer. The calculation results show that the substation capacity load ratio in the substation capacity sizing scheme considering the influence of the peak–valley TOU price is within a reasonable range, and it is increased compared with the traditional sizing. The results show that the modified sizing schemes considering DSM can enhance the adaptability of the power grid and the flexibility of dispatching.

## 5. Conclusions

This paper establishes a cost–benefit analysis model of DSM participants for a future smart grid with multiple information perception application after taking into account the impact of the peak–valley TOU price in distribution network planning. Analysis in the case study demonstrates that under a reasonable price setting (i.e., appropriate pull-off ratio) in a different time period, the profits of DSM participants will increase after considering the implementation of the dynamic peak–valley TOU price in regional distribution network planning. The proposed method also reflects the necessity of combining DSM with distribution network planning.

Of course, there are some limitations in this study: the cost of implementing the TOU price by power supply company and user is ignored, such as the cost of meter transformation, publicity, and promotion, and overtime subsidies increased by the user due to production plan adjustment. After the relevant data are collected in the future, a more in-depth study of the influence of the fixed price on the balanced relationship between the power supply company and the user due to the implementation of the dynamic peak–valley TOU price will need to be conducted.

In addition, after considering the influence of the peak–valley TOU price in distribution network planning, the planned grid structure will inevitably change, which will further affect the reliability of the power supply. The implementation of the peak–valley TOU price will improve the reliability of the power supply. Therefore, the comprehensive impact of these two aspects on the reliability of the power supply can be systematically studied in the future.

## Figures and Tables

**Table 1:** Load data of a typical Day.

*t*	Load/(MW)	*t*	Load/(MW)	*t*	Load/(MW)
1:00	5120	9:00	7185	17:00	7300
2:00	5345	10:00	7350	18:00	7360
3:00	5210	11:00	7535	19:00	7795
4:00	4915	12:00	6710	20:00	7760
5:00	5445	13:00	6835	21:00	7300
6:00	5670	14:00	6935	22:00	6820
7:00	5970	15:00	6890	23:00	5825
8:00	6445	16:00	6870	24:00	5545

**Table 2:** Long-term new load forecast.

Land Usage Type	Area/(km^2^)	Plot Ratio	Load Index/(W·m^−2^)	Demand Factor	Load/(MW)
Residence	53.37	1	50	0.6	1601.10
Culture, education, and health	2.32	0.8	60	1	110.20
Municipal Administration	2.22	0.8	50	1	88.38
Industrial	42.83	1	50	1	2140.96
Office	1.72	1	80	1	136.85
Total (simultaneity rate 0.7)	102.46	/	/	/	2854.24

**Table 3:** Performance of different pull-off ratios.

β	$P_{s}/(\mathrm{RMB}/\mathrm{MWh})$	$P_{v}/(\mathrm{RMB}/\mathrm{MWh})$	$L_{\max}/(\mathrm{MW})$	$L_{\min}/(\mathrm{MW})$	$\lambda_{m}$	$(\Delta C_{u}$ $,\;\Delta E_{p})$
0	590.000	155.263	7280	5179	0.066	(−1743.164, −1491.109)
0.25	691.975	182.099	7124	5267	0.086	(−1226.865, −871.614)
0.5	782.093	205.814	6991	5451	0.103	(−829.035, −384.286)
0.75	862.308	226.923	6847	5631	0.122	(−494.547, 49.745)
1	934.167	245.833	6861	5851	0.120	(−153.341, 71.985)
1.25	998.911	262.871	7062	5869	0.094	(33.441, 422.482)
1.5	1057.547	278.302	7293	5485	0.064	(219.358, 489.678)

**Table 4:** Cost, income, and profit changes of each participant.

Paticipants	Cost Change/105 RMB (ΔC)	Income Change/105 RMB (ΔB)	Profit Change/105 RMB (ΔE)
Power supply company	−269.552	−197.567	71.985
User	−153.341	0	−153.341
Whole society	−269.552	0	269.552

**Table 5:** Power demand forecasting and its corresponding modified results considering DSM (MW).

Sizing Scheme	Maximum Load			
	Past Year 3	Future Year 1	Future Year 2	Future Year 3
Traditional sizing	7795	8575	9605	10,645
Modified sizing considering DSM	6861	7546	8453	9368

**Table 6:** 110 kV substation planning and its corresponding modified results considering DSM (MW).

Sizing Scheme	Item	Future Year 1	Future Year 2	Future Year 3
Traditional sizing	New transformer/set	23	31	31
	Capacity/MVA	1449	1901	1940
Modified sizing considering DSM	New transformer/set	21	27	27
	Capacity/MVA	1297	1701	1675

**Table 7:** Comparison of the investment cost.

Planning Scheme (Million Yuan)	Future Year 1	Future Year 2	Future Year 3
Traditional planning	1449	1901	1940
Modified planning considering DSM	1297	1701	1675

**Table 8:** Load balance verification.

Sizing Scheme	Item	Past Year 3	Future Year 1	Future Year 2	Future Year 3
Traditional sizing	Network supply load (MW)	7795	8575	9605	10,645
	Capacity of the main transformer(MVA)	14,410	15,895	17,760	19,700
	Capacity load ratio	1.85	1.85	1.85	1.85
Modified sizing considering DSM	Network supply load (MW)	6971	7668	8589	9493
	Capacity of the main transformer(MVA)	14,410	15,707	17,408	19,083
	Capacity load ratio	2.07	2.05	2.03	2.01

## Data Availability

Not applicable.
